# Multifocal Insulinoma in Pancreas and Effect of Intraoperative Ultrasonography

**DOI:** 10.1155/2015/375124

**Published:** 2015-07-29

**Authors:** Ersin Borazan, Alper Aytekin, Latif Yilmaz, Muhsin Elci, Mehmet Salih Karaca, Selim Kervancioglu, Ahmet Abdulhalik Balik

**Affiliations:** ^1^Department of General Surgery, Gaziantep University, 27310 Gaziantep, Turkey; ^2^Department of Radiology, Gaziantep University, 27310 Gaziantep, Turkey

## Abstract

Insulinoma is the most frequently seen functional pancreatic neuroendocrine tumor. The incidence of multifocal insulinoma is lower than 10%. Its treatment is direct or laparoscopic excision. The present case was examined with the findings of hypoglycemia and hypercalcemia, and as there was high insulin and C-peptide levels the initial diagnosis was insulinoma. The case was investigated in terms of MEN 1. During preoperative screening for localization, there was one focus in the head of the pancreas in the abdominal tomography and two foci in endoscopic ultrasonography. No other focus was detected through intraoperative visual or manual palpation. However, five foci were detected during operation by intraoperative ultrasonography. The relation of masses with the main pancreatic canal was evaluated and they were excised by enucleation method. There was no recurrence during the postoperative 18-month follow-up of the patient. As a result, during treatment for insulinoma, it should be kept in mind that there might be multifocal foci. In all insulinomas, the whole pancreas should be evaluated with intraoperative ultrasonography because none of the current preoperative diagnostic methods are as sensitive as manual palpation of pancreas and intraoperative ultrasonography. The intraoperative detection of synchronous five foci in pancreas is quite a rare condition.

## 1. Introduction

Insulinoma is the most common cause of endogenous hyperinsulinism related hypoglycemia and it is the most frequent functional pancreatic neuroendocrine tumor (NET). The incidence is presumably between one and three in one million in a year [1]. As it is a rarely encountered clinical situation, successful treatment is more probable with experienced surgeons in experienced centers.

About 5%–10% of patients with insulinoma have MEN 1, which should be excluded or included based on history, symptoms, and radiological and biochemical findings [2]. MEN 1 is an inherited autosomal dominant disease in which tumors develop in endocrine organs. Patients classically have primary hyperparathyroidism secondary to four-gland parathyroid hyperplasia (94%), pituitary adenoma (35%), and multiple pancreatic NETs that may be malignant (75%) [3].

Curative treatment could be achieved by total excision. However, at the present time, there are even cases in which localization could not be precisely determined, in spite of several preoperative tests. This condition increases recurrence, morbidity, and mortality.

The present case is a very rare case in which five synchronous foci were detected in the pancreas. Additionally, the present case emphasizes the fact that intraoperative ultrasonography (IOUS) still plays an important role in insulinoma and multifocal localization should be kept in mind in all patients.

## 2. Case Presentation

The case was admitted to the hospital with complaints of crying and seizures that had persisted for one year. The intellectually disabled, 20-year-old woman had hypoglycemia and hypercalcemia in the biochemical analysis. Her complaints were thought to be related to neuroglycopenic symptoms. As Whipple's triad was clinically detected, further tests were performed with the initial diagnosis of insulinoma. Furthermore, investigations related to the parathyroid and hypophysis glands were performed to exclude MEN 1.

**Figure 1:**
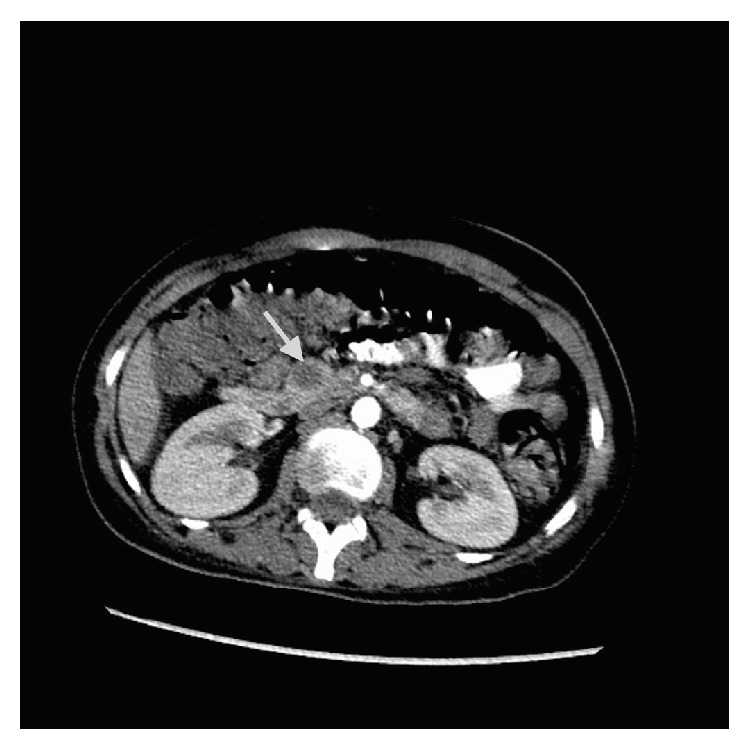
A hypodense mass lesion in the head of the pancreas.

The insulin and C-peptide levels were high (Table 1). In the abdominal CT, there was slight hypodense mass lesion, sized 13 × 12 mm in the head of the pancreas, which had slight peripheral contrast enhancement after the intravenous injection of contrast material (Figure 1). In the endoscopic US, there were hypoechoic, homogenous nodular lesions, 22 mm in size in the head of the pancreas and 7 mm in size in the body of the pancreas. As there was hypercalcemia and increased parathormone levels, we suspect hyperparathyroidism as a part of MEN1 syndrome. So we depended on the neck ultrasonography and the parathyroid scintigraphy to find any lesion in the parathyroid gland, but the radiological investigations were totally normal. The hypophysis MRI was normal too. The patient has been still followed up in terms of hyperparathyroidism.

**Figure 2:**
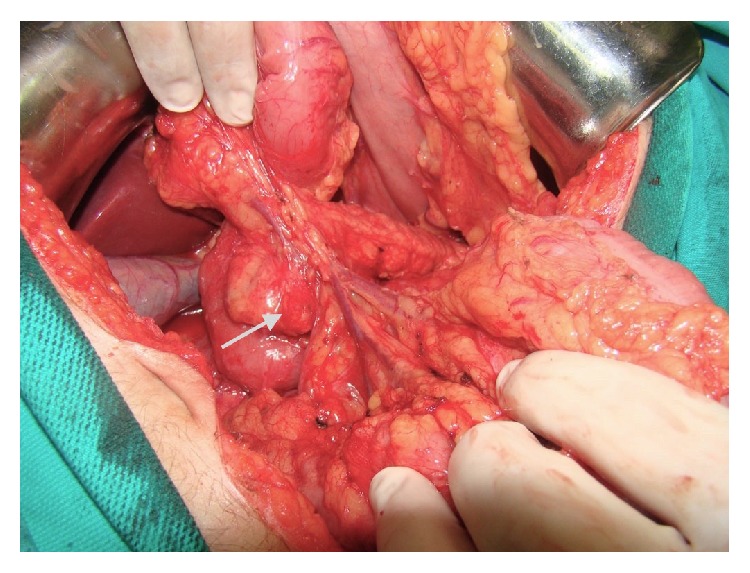
Intraoperatively on palpation, 1 cm nodular lesion in the head of the pancreas.

**Figure 3:**
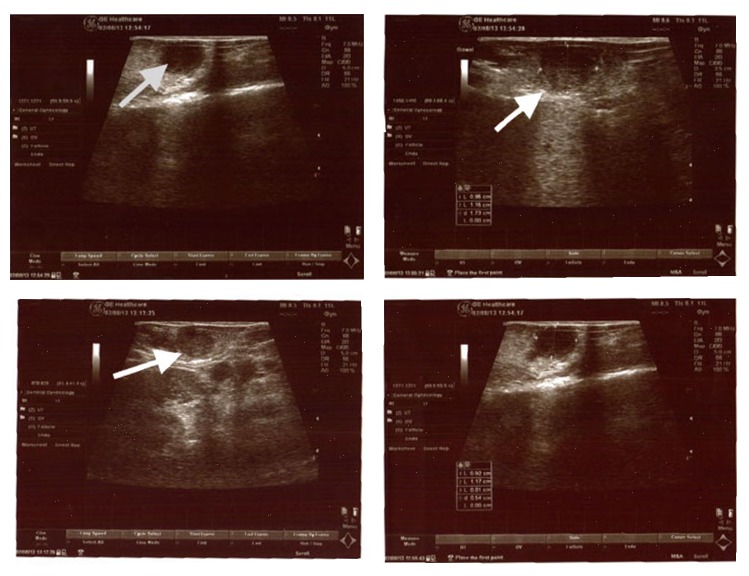
Intraoperative ultrasonography images.

Surgery was planned for insulinoma. Following a midline laparotomy under general anesthesia, the pancreas was explored. On palpation, 1 cm nodular lesions were detected in the head and body of the pancreas (Figure 2). In addition, IOUS was performed, accompanied by a radiologist. Three additional lesions that were not detected during the preoperative period were detected in IOUS (Figure 3).

**Figure 4:**
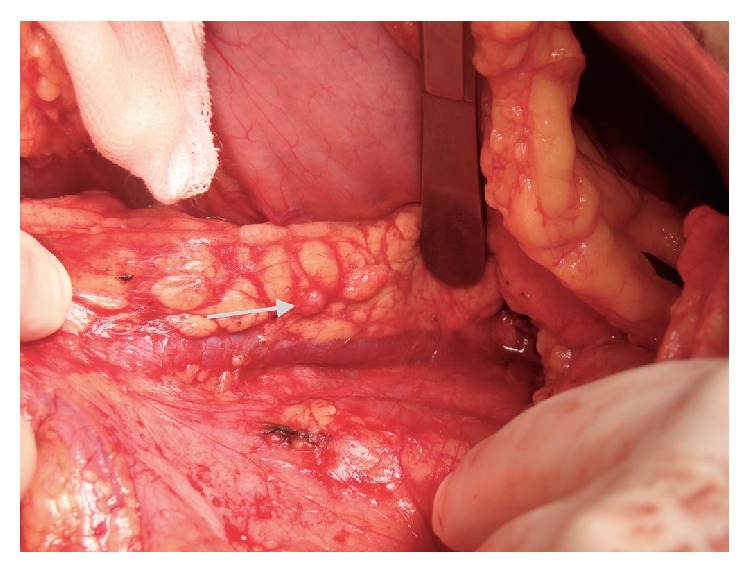
A nodular lesion in the body of the pancreas.

**Figure 5:**
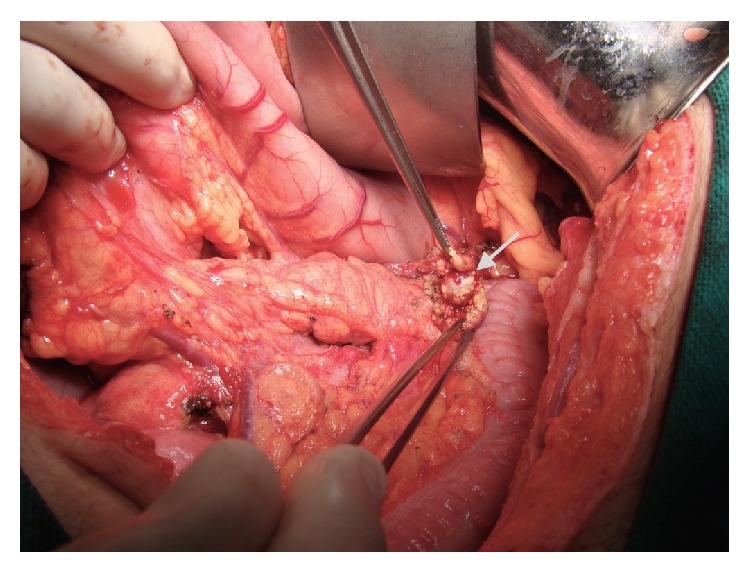
A nodular lesion in the tail of the pancreas.

**Figure 6:**
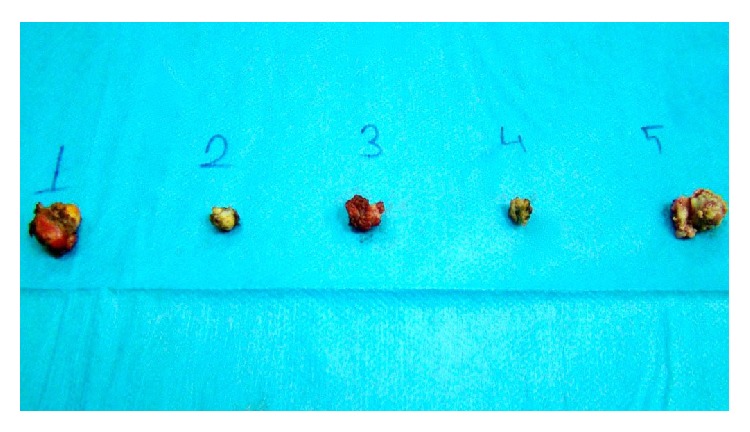
The all pathological lesions after enucleations.

There were a total of five lesions, one in the head of the pancreas, three in the body of the pancreas, and one in the tail of the pancreas, all of which were hypoechoic and with homogenous smooth borders, with the largest one being 1 cm in diameter (Figures 4-5). The sizes of all lesions and their relation with the Wirsung canal were all evaluated. The lesions were removed by enucleation (Figure 6), as they were not closer than 5 mm to the Wirsung canal. Intraoperatively we investigated the glucose levels after the resections of the insulinomas, and we found that it was normal. Intraoperatively the evaluation of insulin levels needs a time, so intraoperatively we could not wait to evaluate the insulin levels after the resections.

The postoperative glucose levels (~85 mg/dL), the insulin level (10,36 *μ*IU/mL), and the C-peptide level (1,82 ng/mL) were normal, and the patient was discharged on the third day. Pancreatic fistulas did not develop in the patient.

The pathologies of all lesions were grade I, well-differentiated, pancreatic NET. The number of mitosis was 1, Ki67 proliferation index was 1%, and there were no vascular or perineural invasions.

During the 18-month follow-up, the patient was in remission. Intermittent endocrinological follow-up of the patient continues in terms of parathyroid adenoma.

## 3. Discussion

Insulinoma is a pancreatic NET, which is generally benign and rarely malignant and can be cured with accurate diagnosis and treatment. It is commonly present in the pancreas; however, its localization within the pancreas might occasionally produce problems. When the tumor is smaller than 1 cm, preoperative and intraoperative localization of the tumor might be difficult [4]. In small tumors that are not close to the pancreas capsule, it is difficult to detect them by visual or palpation.

In our clinic, imaging methods such as abdominal CT, MR, and endoscopic US are preferred in the preoperative screening of insulinoma for localization. Among these, the sensitivity and specificity of endoscopic US are slightly higher. However, its sensitivity also decreases towards the tail. Different methods such as radionuclide scintigraphy, transhepatic portal venous sampling, and selective angiography could be chosen in cases in which localization could not be detected. There is no exact opinion related to the ideal imaging method in the preoperative radiological localization of pancreatic NET. The sensitivity of multislice CT in the detection of pancreatic NET ranges between 71% and 82%. At the present time, recommended CT type by most authors is biphasic or triphasic pancreas visualization [5]. At the same time, CT is useful in the evaluation of metastatic lesions in malignant insulinoma.

In insulinoma cases, the experience in pancreatic surgery and bimanual palpation of the whole pancreas are important. The use of intraoperative manual palpation together with IOUS increases the sensitivity for the localization of insulinoma [6, 7]. Furthermore, the sensitivity and specificity of IOUS during laparoscopic surgery are higher when compared with preoperative imaging techniques [8, 9]. In suitable cases, if laparoscopic US would be possible, laparoscopic methods could be preferred. Furthermore, it has been demonstrated that it is easier to detect multifocal localizations with IOUS when compared with preoperative imaging methods [8].

It is important to use IOUS for both the possibility of multiple lesions and also for investigating the relationship of the mass with the Wirsung canal to choose the surgical method. This method is both an easily applied method and of low cost. Especially in cases with MEN syndrome, the risk of multiple foci and malignancy should be considered.

Correnti et al. [10] reported that IOUS demonstrates the exact localization in all cases. In a double-centered retrospective study, Roland et al. [11] mentioned that the success rate of IOUS was 96.7%. Furthermore, it has also been mentioned that successful treatment with IOUS has been performed in a case in which preoperative localization could not have been detected. However, no cases with multifocal insulinoma have been reported.

In the present case, the preoperative localization of the two lesions was detected with abdominal CT and endoscopic US. However, the three lesions that were found during the intraoperative period have not been detected preoperatively. These lesions could not have been detected visually or by manual palpation. However, it is obvious that the detection of these unseen foci by imaging with IOUS prevents possible recurrences and complications. If IOUS had not been used, these three foci would not have been recognized.

The IOUS is also successful in a false nodular adenoma. Thus, unnecessary resection and the risk of fistula could be decreased.

One of the important factors that should be mentioned here is the surgical treatment technique. According to the Japanese GEPNET clinical guideline, in lesions that are 2 cm or smaller and in lesions that are more than 3 mm away from the main pancreatic duct, enucleation is recommended rather than pancreatic resection [12]. In the present case, enucleation was performed on all lesions, as the lesions were 5 mm away from the main pancreatic canal according to IOUS.

In conclusion, curative surgical treatment with IOUS was performed in the present case and her follow-up continues without any problems.

## Figures and Tables

**Table 1:** The patient preoperative laboratory values.

Parameters	Results	Reference range
Fasting glucose	58 mg/dL	74–109
Insulin	*167.9 μIU/mL *	3–25
C-peptide	*141 ng/mL *	0.9–7.1
Gastrin	12.9 pg/mL	40–210
ACTH	24.7 pg/mL	10–46
Growth hormone	3.92 ng/mL	0.05–8.6
Cortisol	19.19** ** *μ*g/mL	3.7–19.4
TSH	1.95 *μ*IU/mL	0.27–4.2
Calcium	*11.2 mg/dL *	8.5–10.5
Parathormone	*156 pg/mL *	14.9–56.9
